# Unbeneficial effects of not prescribing antibiotics to pediatric patients with acute upper respiratory infection: a descriptive epidemiological study based on a large Japanese medical claim database

**DOI:** 10.1186/s40780-025-00519-1

**Published:** 2025-12-08

**Authors:** Kanako Mizuno, Ryo Inose, Yuichi Muraki

**Affiliations:** Laboratory of Clinical Pharmacoepidemiology, Kyoto Pharmaceutical University, Kyoto, 607-8414 Japan

**Keywords:** Medical claim database, Acute upper respiratory infection, Antimicrobial stewardship

## Abstract

**Background:**

National Action Plan on Antimicrobial Resistance in Japan recommends further strengthening of antimicrobial stewardship (AS) for acute upper respiratory infection (URI) in outpatients. AS initiatives for outpatients include the establishment of an AS implementation fee that can be claimed when a physician does not prescribe antibiotics to a pediatric patient diagnosed with acute URI that does not require antibiotics, after providing sufficient explanation. However, in Japan, unbeneficial effects of not prescribing antibiotics for acute URIs have not been clarified. This study aimed to investigate whether there were any unbeneficial effects in pediatric patients with acute URIs who claimed the AS implementation fee and were not prescribed antibiotics using a large Japanese medical insurance claim database.

**Methods:**

This study used a large Japanese medical insurance claim database provided by IQVIA Japan. Patients aged less than six years of age and with a definitive diagnosis of acute URI and who claimed the AS implementation fee from January 2019 to December 2021 were selected. Among these patients, those with prescriptions other than antibiotics on the date of the first definitive diagnosis of acute URI were included in this study. The prescription of medicines and hospitalization within 10 days of the date of the first definitive diagnosis of acute URI in the target patients were investigated.

**Results:**

There were 967,546 patients with a definitive diagnosis of acute URI in the outpatient. Of these, 32,489 patients below six years of age who claimed the AS implementation fee for children were prescribed medications other than antibiotics were considered the target patients for this study. Of these, 12,101 (37.2%) were again prescribed drugs in the outpatient clinic within 10 days, and 2,275 (7.0%) were prescribed antibiotics. The median (interquartile range) number of days until antibiotics were prescribed was 4 (2–7). Additionally, 105 patients (0.3%) were hospitalized within 10 days.

**Conclusion:**

This study revealed that there may be at least one risk factor in patients with acute URIs who were not prescribed antibiotics. In case of acute URI diagnosis and absence of antibiotic prescription, patients should be warned of worsening symptoms for at least 4 days.

**Supplementary information:**

The online version contains supplementary material available at 10.1186/s40780-025-00519-1.

## Background

Approximately 90% of acute upper respiratory infections (URIs) are caused by viruses. However, antibiotics are prescribed for viral URIs that do not require antibiotics in outpatients [1]. In a Japanese survey conducted from 2012 to 2017, antibiotics were prescribed to approximately 32% cases of acute respiratory tract infections that did not require antibiotics; moreover, approximately 90% of these antibiotics were broad-spectrum antibiotics [2]. Additionally, a survey of preschool children revealed that antibiotics had been prescribed for 66.4% of children [3]. The National Action Plan for Antimicrobial Resistance (AMR) 2023–2027, revised in Japan in April 2023, recommends further strengthening of antimicrobial stewardship (AS) for URIs in outpatients [4].

One such Japan’s initiatives for URI is the Manual of AS, issued in 2017 and continually revised [5]. In the first edition, the treatment policy was only for children of school age and older and adults; the 2019 revision added a treatment policy for children younger than school age [5, 6]. The recommended treatment strategy for children younger than school age is not to administer antibiotics or antibiotic prophylaxis [6]. However, even if the first diagnosis is common cold or acute sinusitis, antibiotics may be indicated when the patient’s respiratory status worsens.

Another AS initiative for outpatients is the AS implementation fee for pediatric patients with acute URIs. This reimbursement was established in 2018, and eligibility was raised in 2020 from below three years to below six years of age [7]. A fee of 800 yen (approximately US$5.3) can be claimed when a physician diagnoses a pediatric patient with acute URI or acute diarrhea, determines that antibiotics are not necessary, provides sufficient explanation to the patient, and does not prescribe antibiotics. Previous reports have shown that the prescription and use of total antibiotics, broad-spectrum antibiotics, and symptomatic drugs, such as expectorants, decreased after the introduction of the AS implementation fee for pediatric patients [8].

Thus, various countermeasures have been implemented in Japan to avoid prescribing antibiotics for patients with acute URIs. However, acute bacterial sinusitis has been reported to complicate approximately 8% of acute URIs in pediatric patients [9]. Therefore, antibiotics may be required after the diagnosis of acute URI. However, in Japan, the unbeneficial effects of not prescribing antibiotics for acute URIs have not been clarified.

This study aimed to investigate whether there were any unbeneficial effects of not prescribing antibiotics to pediatric patients with acute URIs who claimed AS implementation fees using a large Japanese medical insurance claim database.

## Methods

### Data source

This study used a large Japanese medical claims database provided by IQVIA Japan. This database is based on information collected from health insurance in Japan and included data for approximately 3.3% of the total Japanese population as of the fiscal year 2021. In addition, this database contained only International Classification of Diseases, 10th edition codes A00–B99, H60–H95, J00–J99, L00–L99, and N00–N99 related to injuries and diseases.

### Study period and patient selection

The study was conducted from January 2019 to December 2021. Children below six years of age who claimed the AS implementation fee for pediatric patients with acute URI at the time of definitive diagnosis of acute URI were selected. Patients with prescriptions other than antibiotics were included in this study. Patients without prescription information on the date of the first definitive diagnosis of acute URI were also excluded because it was not possible to determine whether the patients actually had no prescription or had missing prescription data.

### Definition

Among patients with a definitive diagnosis of acute URI (Japanese disease code: 4659007), those who obtained an AS fee for pediatric patient (Japanese medical practice codes: 113024670, 113027870) with the same Claim ID Number as the definitive diagnosis of acute URI were defined as those with acute URI for which an additional fee for AS was calculated. The date the AS implementation fee reimbursement claimed was defined as the date of the first definitive diagnosis of acute URI. Furthermore, hospitalization information was extracted using a flag indicating whether the visit was inpatient or outpatient, assigned to the Claim ID Number within the database. In this study, drugs with Anatomical Therapeutic Chemical (ATC) code, A07AA, J01A, J01B, J01C, J01D, J01E, J01F, J01G, J01M, J01R, and J01X were defined as antibiotics. J01DD, J01FA, and J01MA were defined as broad-spectrum antibiotics [2]. Based on the guidelines for AS promotion [5], the prescription of medications and hospitalization within 1–10 days of the first definitive diagnosis of acute URI was defined as a return visit due to worsening of acute URI. Furthermore, if a claim with a different Claim ID Number than the one used to claim the AS implementation fee was identified from a medical institution on the same date the patient received their first definitive diagnosis of acute URI, the patient was considered having visited multiple medical institutions.

### Statistical analysis

The status of return visit within 1–10 days of the date of the first definitive diagnosis of acute URI was investigated. In addition, the disease and drug prescription status of patients who made return visit were surveyed. Sensitivity analysis included patients without prescription information on the date of first definitive diagnosis of acute URI to clarify the prescription rates of medications, antibiotics, as well as hospitalization rates within 10 days of the date of first definitive diagnosis of acute URI. Stata version 18.0 (Stata Corp LLC, College Station, TX, USA) was used for the analysis.

### Ethical consideration

This study was approved by the Ethics Committee of Kyoto Pharmaceutical University (approval number: E-00031). The requirement for informed consent was waived owing to the anonymized nature of the study data.

## Results

### Patient selection

**Fig. 1:**
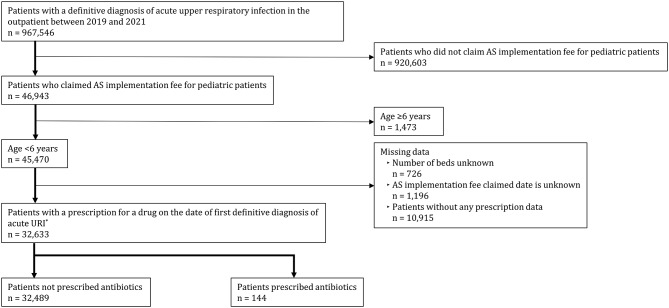
Flowchart of patient selection. *In this study, the date the AS implementation fee reimbursement claimed was defined as the date of the first definitive diagnosis of acute URI

Patient selection flowchart is shown in Fig. 1. There were 967,546 patients with a definitive diagnosis of acute URI in the outpatient department. In addition, 45,470 patients were less than six years old, for whom the AS implementation fee for pediatric patients was claimed at the time of the first definitive diagnosis of acute URI. There were 32,633 patients who had prescription for medications on the date of the first definitive diagnosis of acute URI. Of these, 32,489 patients who were not prescribed antibiotics on the date of their first definitive diagnosis of acute URI were included in this study.

On the other hand, 144 (0.4%) patients were prescribed antibiotics on the date of their first definitive diagnosis of acute URI. Among these, 56 (38.9%) had only visited medical institutions that claimed the AS implementation fee on the date of their first definitive diagnosis of acute URI. The remaining 88 (61.1%) patients visited a different medical institution on the date of their first definitive diagnosis of acute URI than the institution where the AS implementation fee was claimed.

### Patient characteristics

The background characteristics of the 32,489 patients are presented in Table 1. Overall, 76.9% were 0–2 years old, with no difference between the sexes. In addition, 98.9% of the patients had fewer than 20 beds. There were 12,975 (39.9%) patients who had only acute URI at the time of the first definitive diagnosis. More than 60% of the patients had multiple diagnoses, such as acute bronchitis, eczema, and allergic rhinitis, diagnosed at the same time as acute URI.

**Table 1:** Patient characteristics of 32,489 target patients

	n (%)
**Sex**	
Man	16,424 (50.6)
Woman	16,065 (49.5)
**Age**	
0	5,209 (16.0)
1	12,289 (37.8)
2	7,491 (23.1)
3	3,695 (11.4)
4	2,022 (6.2)
5	1,783 (5.5)
**Bed capacity**	
< 20	32,129 (98.9)
20–99	92 (0.3)
100–499	262 (0.8)
≥500	6 (0.02)
**Patients diagnosed with only acute URI**	12,975 (39.9)
Name of the infectious disease claimed with the same Claim ID Number as acute URI^*1^	
Acute bronchitis	6,635 (20.4)
Eczema	3,197 (9.8)
Allergic rhinitis	3,066 (9.4)
Asteatosis	2,184 (6.7)
Acute gastroenteritis	1,496 (4.6)
**Patients prescribed antibiotics within 10 days**	2,275 (7.0)
**Days until antibiotics were prescribed (days)**	4 (2–7)^*2^
**Patients hospitalized within 10 days**	105 (0.3)

^*1^ The five most frequent diseases claimed with the same Claim ID number as acute upper respiratory infection was claimed

^*2^ Data shows median (interquartile range)

### Prescription status in outpatient clinic within 10 days of the date of the first definitive diagnosis of acute URI

A total of 12,101 (37.2%) patients revisited the clinic within 10 days of the first definitive diagnosis of acute URI and were again prescribed medications. Of these, 2,275 (7.0%) were prescribed antibiotics within 10 days. The median (interquartile range) number of days until antibiotics were prescribed was 4 (2–7) (Table 1). Approximately 55% of patients were treated with broad-spectrum antibiotics (Table 2).

**Table 2:** Details of antibiotics prescriptions for 2,275 patients who were prescribed antibiotics within 10 days

ATC 4		Oral	Injectable	All
J01CA	Penicillins with extended spectrum	338.9	0.0	338.9
J01DD	Third-generation cephalosporins	282.2	2.6	284.8
J01FA	Macrolides	217.6	0.0	217.6
J01MA	Fluoroquinolones	87.5	0.0	87.5
J01CR	Combinations of penicillins, including beta-lactamase inhibitors	68.6	0.0	68.6
J01DH	Carbapenems	19.8	0.0	19.8
J01XX	Other antibacterials	14.5	1.3	15.8
J01GB	Other aminoglycosides	0.0	15.4	15.4
J01DC	Second-generation cephalosporins	9.2	0.0	9.2
J01DB	First-generation cephalosporins	7.9	0.0	7.9
J01DI	Other cephalosporins and penems	3.1	0.0	3.1
J01FF	Lincosamides	0.0	2.6	2.6
J01EE	Combinations of sulfonamides and trimethoprim, incl. derivatives	2.2	0.0	2.2
A07AA	Antibiotics	1.3	0.0	1.3
J01AA	Tetracyclines	1.3	0.0	1.3
J01XB	Polymyxins	1.3	0.0	1.3

Data are expressed as the number of prescriptions/1,000 patients

The denominator is 2,275 patients who were prescribed antibiotics within 10 days

ATC: Anatomical Therapeutic Chemical

### Hospitalization status within 10 days of the date of the first definitive diagnosis of acute URI

A total of 105 (0.3%) patients were hospitalized within 10 days of the date of the first definitive diagnosis of acute URI. The background characteristics of the 105 patients are presented in Table 3. There were 41 patients who were prescribed antibiotics within 0–10 days of hospitalization. Some patients who were prescribed antibiotics within 0–10 days of hospitalization were diagnosed with bacterial diseases.

**Table 3:** Patient characteristics of 105 patients hospitalized within 10 days

	n (%)
**Sex**	
Man	51 (48.6)
Woman	54 (51.4)
**Age, years**	
0	32 (30.5)
1	40 (38.1)
2	19 (18.1)
3	9 (8.6)
4	3 (2.9)
5	2 (1.9)
**Infectious disease name in 41 patients for whom antibiotics were prescribed within 10 days of hospitalization**^**1**^	
Urinary tract infection	16 (39.0)
Acute pneumonia	4 (9.8)
Acute bronchitis	3 (7.3)
Respiratory syncytial virus infection	2 (4.9)
Pharyngitis	2 (4.9)

^*1^ The five most frequently claimed infectious diseases at the time of hospitalization

### Sensitivity analysis

Patient selection flowchart for sensitivity analysis is shown in Supplemental Fig. 1. The sensitivity analysis involved 43,404 patients, including 10,915 patients without prescription information on the date of first definitive diagnosis of acute URI. Furthermore, 14,424 (33.2%) patients were prescribed medication again within 10 days of the date of first definitive diagnosis of acute URI. Among these, 2,759 (6.4%) were prescribed antibiotics. Additionally, 169 (0.4%) patients were hospitalized within 10 days of the date of first definitive diagnosis of acute URI.

### Discussion

This study investigated, for the first time to the best of our knowledge, the unbeneficial effects of not prescribing antibiotics to patients with acute URI who claimed the AS implementation fee for pediatric patients. This study revealed, for the first time that 37.2% of patients with acute URI who claimed the AS implementation fee were again prescribed a drug within 10 days, and 7.0% were prescribed antibiotics within 10 days. In addition, the median (interquartile range) time to antibiotic prescription was 4 (2–7) days. The Manual of AS state that antibiotics should be considered when worsening or prolonged symptoms occur [6]. Therefore, it is considered possible that a new bacterial complication may have occurred after acute URI [9, 10]. In addition, acute respiratory tract infections in infants are generally considered difficult to accurately assess for symptoms and signs [6]. As a result, the patient may have already had a complication of bacterial infection at the time of first diagnosis, but the symptoms were missed. This suggests that even if the decision is made not to prescribe antibiotics at the first visit, it is necessary to monitor worsening of symptoms for at least 4 days.

In other countries, various approaches to AS are being adopted by health insurance pharmacies, including point-of-care white blood cell counts at first diagnosis and follow-up after 48 h [11]. In the United Kingdom, delayed prescription is used in approximately one-third of patients with cough as the main symptom [12]. Education for prescribers and feedback on antibiotic prescription have been shown to contribute to the optimization of antibiotics for viral URIs, according to a study conducted in the United States [13]. In Japan, AS is practiced in hospitals but not considerably in clinics, unlike other countries. However, in Japan, the usefulness of long-term follow-up by a pharmacist via telephone in the management of side effects of oral anticancer drugs has been reported in a recent study [14]. Thus, it was considered necessary to establish a system whereby pharmacists could provide information and follow-up on possible worsening of acute URIs in patients not prescribed antibiotics.

In surveys prior to 2017, third-generation cephalosporins were primarily prescribed for children [2]. However, in our study, penicillin was most frequently prescribed. This change suggests that the development of the National Action Plan on AMR and the Manual of AS may have influenced prescribing choices. Amoxicillin, the most frequently prescribed antibiotic in this study, is recommended in the manual as the first-line treatment for Group A β-hemolytic *Streptococcus* pharyngitis and acute otitis media [6]. Additionally, macrolide antibiotics are recommended drugs for specific infections such as pertussis and *Mycoplasma* bronchitis [6]. Broad-spectrum antibiotics such as third-generation cephalosporins and fluoroquinolones—which are not typically recommended for bacterial complications of acute URI—are still being prescribed in a few cases. Although the shift toward first-line drugs is progressing, the continued prescription of certain broad-spectrum antibiotics remains a critical challenge for the appropriate use of antibiotics in the future.

This study revealed that 144 (0.4%) patients were prescribed antibiotics on the same day the AS implementation fee was claimed. Among these patients, 61.1% had visited a different medical institution on the date of their first definitive diagnosis of acute URI than the institution where the AS implementation fee was claimed. Therefore, patients may have received treatment at multiple medical institutions, and antibiotics were likely prescribed at a facility different from the one that claimed the AS implementation fee. However, information about other institutions that claimed the AS implementation fee was unavailable for 38.9% of patients, and antibiotics may have been prescribed at the medical institution where the AS implementation fee was claimed. These results suggest that the current application of this additional fee may be insufficient to achieve its intended purpose. Therefore, to truly promote the appropriate use of antibiotics, stricter adherence to calculation requirements and improvements to the monitoring system may be necessary.

In this study, a sensitivity analysis was conducted on a cohort of 10,915 patients for whom no prescription drug information was available on the date of first definitive diagnosis of acute URI. The results showed that 14,424 (33.2%) patients were prescribed medications again within 10 days of the date of first definitive diagnosis of acute URI, and 2,759 (6.4%) patients were prescribed antibiotics. Additionally, 169 (0.4%) patients were hospitalized within 10 days of the date of first definitive diagnosis of acute URI. These results were largely consistent with the main analysis, supporting the robustness of the findings.

This study had few limitations. First, each medical institution in Japan submits insurance claims on a per-patient and per-month basis; therefore, the date of the diagnosis claim is unknown. Second, the database used in this study does not reflect Japan as a whole, and therefore, may not reflect the actual situation in Japan. Third, this study is based on data from 2019 to 2021, exhibiting a temporal limitation, as it does not capture subsequent changes in the healthcare environment. While this was the most recent data available at the start of the research, continuous evaluation is desirable to accurately reflect recent trends. Fourth, this study defines diseases solely based on Japanese disease codes, which may overestimate the actual number of patients. In clinical practice, disease names may be recorded for administrative purposes, such as examinations or prescriptions, without a definitive diagnosis [15]. This represents a limitation of the study, as the database does not allow distinction between diagnoses recorded as definitive and those assigned procedural reasons. Despite these limitations, the findings of this study provide useful information for the future AS promotion in outpatients.

## Conclusions

This study revealed the possibility of at least one risk, such as the prescription of antibiotics or hospitalization, in patients with acute URIs for whom antibiotics were not prescribed. In case of acute URI diagnosis and absence of antibiotic prescription, patients should be warned of worsening symptoms for at least 4 days. A follow-up system for patients with acute URIs for whom antibiotics are not prescribed needs to be developed.

## Electronic supplementary material

Below is the link to the electronic supplementary material.

Supplementary Material 1

## Data Availability

No datasets were generated or analysed during the current study.

## Abbreviations

AMR: Antimicrobial Resistance
AS: antimicrobial stewardship
ATC: Anatomical Therapeutic Chemical
URI: upper respiratory infection
