# Supplementary material for: Unbeneficial effects of not prescribing antibiotics to pediatric patients with acute upper respiratory infection: a descriptive epidemiological study based on a large Japanese medical claim database
Source: J Pharm Health Care Sci. 2025 Dec 8;12:4. doi: 10.1186/s40780-025-00519-1 (PMC12797695; doi:10.1186/s40780-025-00519-1)
Supplement: Supplementary file 1 — Supplementary Material 1 [file 40780_2025_519_MOESM1_ESM.pptx]

## Slide 1
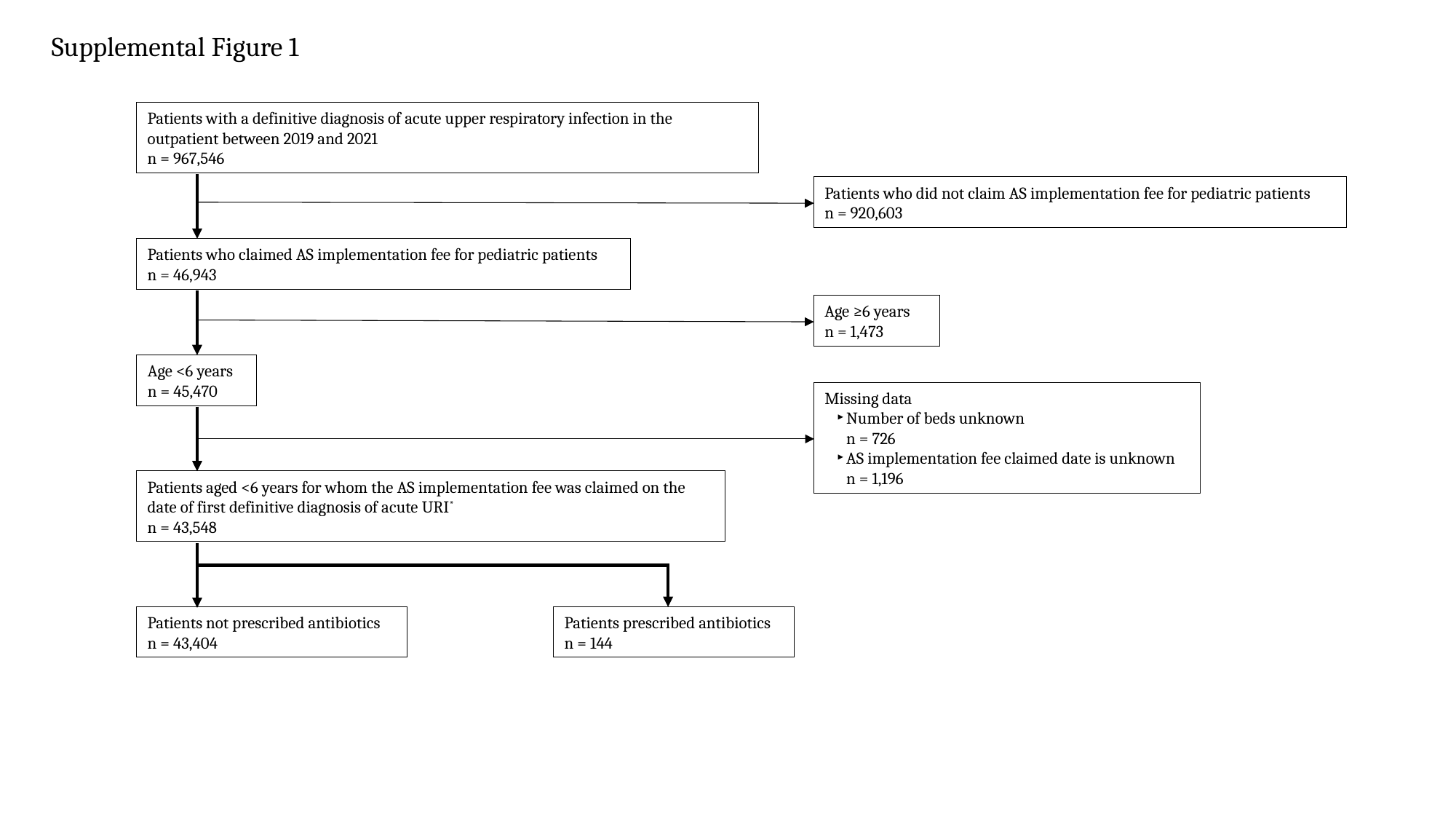

Supplemental Figure 1
Patients with a definitive diagnosis of acute upper respiratory infection in the outpatient between 2019 and 2021
n = 967,546
Patients who did not claim AS implementation fee for pediatric patients
n = 920,603
Patients who claimed AS implementation fee for pediatric patients
n = 46,943
Age ≥6 years
n = 1,473
Age <6 years
n = 45,470
Missing data
Number of beds unknown
n = 726
AS implementation fee claimed date is unknown
n = 1,196
Patients aged <6 years for whom the AS implementation fee was claimed on the date of first definitive diagnosis of acute URI*
n = 43,548
Patients not prescribed antibiotics
n = 43,404
Patients prescribed antibiotics
n = 144
